# The Effects of the Dynamic Thermophysical Properties of Clothing and the Walking Speed Input Parameter on the Heat Strain of a Human Body Predicted by the PHS Model

**DOI:** 10.3390/ijerph17186475

**Published:** 2020-09-05

**Authors:** Qianqian Huang, Jun Li

**Affiliations:** 1 College of Fashion and Design, Donghua University, Shanghai 200051, China; 18354287639@163.com; 2 Key Laboratory of Clothing Design and Technology (Donghua University), Ministry of Education, Shanghai 200051, China

**Keywords:** PHS model, heat strain, heat stress, clothing insulation, clothing dynamic insulation

## Abstract

The prediction accuracy of the Predicted Heat Strain (PHS) model is affected by the correction approaches of static thermophysical properties of clothing considering the pumping effects of wind and body movement. In this study, a comparison of different correction algorithms for three types of clothing and their influence on the heat strain predicted by the PHS model was carried out with experimental data obtained from the literature. Results show that the dynamic insulation values calculated by ISO 9920 corrections are larger than those obtained by ISO 7933 when the static insulation values are higher than 0.4 clo, but when the static values are lower than 0.4 clo, it varies contrarily. The dynamic evaporative resistance values calculated with ISO 9920 equations are larger than those with ISO 7933. The prediction accuracy of the PHS model with ISO 9920 corrections and the addition of the walking speed input parameter can be improved for normal clothing (NC) in a hot environment and high clothing insulation. For specialized, insulating, cold weather clothing (SC), ISO 7933 corrections with an added walking speed input parameter to the PHS model have a good prediction precision.

## 1. Introduction

Heat strain is usually faced by people in various occupations, such as firefighters, cooks, construction workers, and soldiers. It has an impact on the work efficiency, the productivity, and may even threaten life due to the risk of heat disorders that can be affected by the metabolic rate, hot environments, or clothing factors.

The Predicted Heat Strain (PHS) model may be the most feasible index examined for the assessment of the potential health problems related to physiological strain [1], and it is specified by ISO 7933-2004 [2]. The PHS model is based on human body heat balance equations and can be used to predict minute-by-minute rectal temperatures and sweat rates. The validation of the PHS model contained data from 672 experiments in laboratories and 237 experiments in the field [3]. PHS with ISO 7933 used dynamic clothing insulation for the prediction of heat exchanges between a clothed person and the environment considering the influence of the wind and body movement, and the correction approach of clothing insulation was proposed by Holmér [4]. However, Wang [5,6] found that PHS with ISO 7933 overestimated rectal temperature by 1.8 °C at the end of 70 min of exercise when in firefighting gear, and speculated that the reason could be related to the correction approach of the thermophysical properties of clothing. Havenith [7] reorganized the experimental data, and then divided the clothing into three types: normal clothing, light clothing, and cold weather clothing, and then partly proposed the new correction formulas, which were adopted by ISO 9920 [8]. Unfortunately, the correction approaches of static clothing insulation were significantly different between ISO 7933 and ISO 9920 [9]. Moreover, some further correction approaches for light clothing were devoted to quantifying the dynamic thermophysical properties of clothing instead of regarding it as the intermediate state between normal clothes and nudity, which ISO 7933 and ISO 9920 applied [10].

Some researchers have already begun to investigate the effect of the correction approaches of the thermophysical properties of clothing on the predicted precision of the PHS model. Long [11] found that using ISO 9920 to modify the clothing algorithm in the PHS model would shorten the forecast maximum heat exposure time and improve protection efficiency. Alfano [9] also found that PHS with the ISO 9920 correction formula would reduce the maximum exposure time by 3–6 h, so it was considered that the ISO 9920 correction formula could not be directly introduced into the calculation of the PHS model, but a new correction was needed. However, different clothing types and their correction formulas were not treated separately, and some further correction approaches were not involved.

Furthermore, most research suggested six factors [1,12,13] (air temperature, *Ta*; partial water vapor pressure, *Pw*; air velocity, *v_a_*; heat radiation, *Tr*; metabolic rate, *Met*; and clothing) needed for the appropriate assessment of heat strain. Although walking speed, *v_w_*, is an essential parameter for calculating the dynamic thermophysical properties of clothing, it was considered an intermediate variable determined by the metabolic rate. There is no evidence showing whether adding *v_w_* could improve PHS prediction accuracy.

In conclusion, the objective of this study is to investigate the effects of the correction approach of thermophysical properties of clothing and the input parameter *v_w_* on the heat strain level predicted by the PHS model and determine the application guidelines for three clothing types.

## 2. Materials and Methods

In order to meet the objective, this study was divided into two parts. The first part compared the correction approaches of the thermophysical properties of clothing and calculated the values of dynamic clothing insulation and dynamic evaporative resistance. The second part studied the effects of the correction approaches of clothing and the input parameter *v_w_* on the heat strain level by comparing predicted values with human exposure experiment data.

### 2.1. Correction of the Thermophysical Properties of Clothing

#### 2.1.1. Correction of Thermal Insulation

Clothing can be divided into three types according to ISO 9920, the correction approaches for the three types of clothing are defined as follows.

Normal clothing (NC: 0.6 clo < *I_cl_* < 1.4 clo): the correction approach for NC is by multiplying the coefficient based on the basic insulation, as in Equations (1)–(5).

(1) $$I_{tot}=I_{cl}+\frac{I_{a}}{f_{{}_{cl}}}$$

(2) $$f_{cl}=1+0.3I_{cl}$$

(3) $$I_{tot,dyn}=C_{orr,tot}*I_{tot}$$

(4) $$I_{a,dyn}=C_{orr,a}*I_{a}$$

(5) $$I_{cl,dyn}=I_{tot,dyn}-\frac{I_{a,dyn}}{f_{cl}}$$

where *I_cl_* is the basic clothing insulation, as an input parameter, clo. *I_a_* is the static boundary layer thermal insulation, about 0.716 clo [2]. *I_tot_* is the total basic clothing insulation, clo. The suffix *dyn* appears to refer to the dynamic conditions. *f_cl_* is the ratio of the outer surface area of the clothed body to the surface area of the nude body. *C_orr_* is the correction coefficient.

Correction coefficients and their ranges are summarized in Table 1. ISO 7933 adopted the result of the study of Holmér [4], which was based on the data of 10 sets of clothing. Later, Havenith [7] rearranged the same experimental data to revise the condition that the correction coefficient was greater than 1, adjusted the relative air velocity (*v_ar_*) in the windless state to 0.15 m/s, and removed the constant term. ISO 9920 used Havenith’s correction formulas to estimate the dynamic thermal resistance (*R^2^* = 0.93).

Specialized, insulating, cold weather clothing (SC: *I_cl_* > 1.4 clo): ISO 9920 used the approach of correction coefficient to correct the thermal insulation, as shown in Table 2. The air permeability parameter was added to increase the prediction accuracy (*R^2^* = 0.968). However, ISO 7933 still used the correction coefficient of NC for SC.

Light clothing (LC: *I_cl_* < 0.6 clo): Table 3 showed the different approaches of corrections. ISO 7933 used the correction coefficient of thermal insulation and the boundary layer of NC to correct for LC, and ISO 9920 used an interpolation approach to correct based on the dynamic clothing insulation of NC and the boundary layer. Neither algorithms directly considered the influence of wind and body movement, but the algorithm from the study of Lu [10] was added.

#### 2.1.2. Correction of Evaporative Resistance

Both ISO 7933 and ISO 9920 provided the correction approaches for dynamic evaporative resistance, as shown in Table 4. ISO 7933 was based on a double correction approach of the thermal insulation and the permeability index. However, ISO 9920 has only one correction coefficient for the evaporative resistance. Therefore, when the ISO 9920 algorithm was used in the PHS model, it needed to increase the static evaporative resistance input parameter.

#### 2.1.3. Predicted Dynamic Clothing Insulation and Evaporative Resistance

Taking into consideration the correction algorithms of clothing thermophysical properties for the three types of clothing, air speed (0.5, 1, 3 m/s), walking speed (0.4, 0.8, 1.2 m/s), and basic clothing insulation (0.2, 0.4, 0.6, 0.8, 1, 1.5, 2 clo) were used to calculate the dynamic thermal insulation and evaporative resistance. For example, the dynamic thermal insulation of NC included two algorithms ISO 7933 and ISO 9920 according to Table 1 and Table 2, and 0.6, 0.8, 1 clo basic clothing insulations were involved.

### 2.2. Exposure Assessment of the PHS Model

According to ISO 7933 E.2 program, we constructed algorithms to compute the PHS model in MATLAB R2016a (Mathworks, Natick, America), which was validated to meet the requirements of Annex F. Then we altered the correction formula of the thermal insulation and the evaporative resistance with the approaches of ISO 9920 or Lu instead of ISO 7933 for the three types of clothing. Basic clothing insulations 0.63, 1.08, and 1.11 clo were used for NC and involved three environmental conditions. As for LC and SC, only one basic clothing insulation 0.48 and 2.01 clo, respectively, for each was used, and the air temperature was 30 and 40 °C. The air velocity was 0.33 m/s and the walking speed was 1.25 m/s for all tests. Table 5 lists the primary input parameters for the PHS model.

The predicted results of rectal temperature, water loss, and maximum exposure time were compared with the experimental data provided from the literature of the study of Wang [5,6]. Six unacclimated male subjects participated in the experiment wearing NC and SC, and their mean height was 1.78 ± 5 m and the mean weight was 80 ± 8 kg. Another eight unacclimated male volunteers, mean height 1.76 ± 0.06 m and the mean weight 77 ± 10.2 kg, took part in the experiment wearing LC. The subjects were all healthy and were informed not to smoke and consume alcohol, coffee, or tea 24 h before the experiment. They were also requested not to do intensive activities for at least 1 h before the experiment. Each subject came to the laboratory and carried out the exposures at the same time of each day. The study procedures followed the Declaration of Helsinki.

### 2.3. Statistical Analysis

SPSS 22.0 was used for statistical analysis, in which paired sample T-test was used to test whether there were significant differences between different algorithms. The level of significance was set at *p* < 0.05.

In order to eliminate the influence of the initial state, such as skin and rectal temperature, the first 10 min of data were removed during statistical analysis. Means and standard deviation (SD) of the experimental data were reported. The root mean square deviation (rmsd), which is the average absolute difference between the results of simulations and the corresponding human experiments, is defined as

(6) $$rmsd=\sqrt{\frac{{\left(x_{measured}-x_{predicted}\right)}^{2}}{n}}$$

where *x_measured_* is the value measured in human subjects, *x_predicted_* is the value predicted by the PHS model, and n is the number of data points in the experiment. The rmsd serves to evaluate the model’s goodness of fit and the bias describes the model’s accuracy [14]. The fit is considered as acceptable when the rmsd is smaller than the SD of the experimental data.

The bias, which is the averaged error, should be equal to or close to zero, or not beyond the SD of the given data set to ensure unbiased model prediction, and it is defined as

(7) $$bias=\frac{\sum \left(x_{measured}-x_{predicted}\right)}{n}$$

## 3. Results

### 3.1. Predicted Dynamic Thermophysical Properties of Clothing

#### 3.1.1. Clothing Dynamic Thermal Insulation

**Figure 1:**
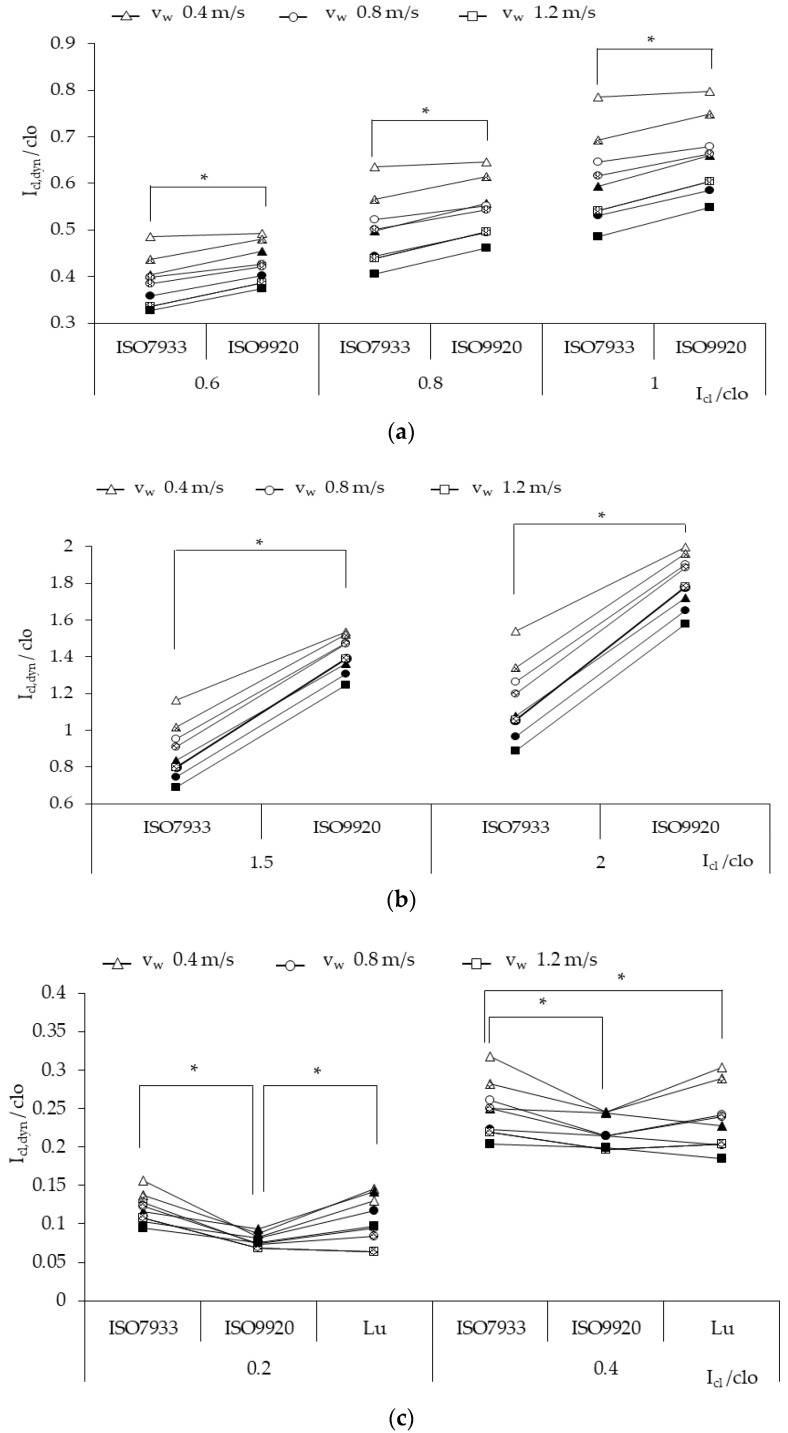
The dynamic clothing insulation of three types of clothing: (**a**) NC, (**b**) SC, (**c**) LC, calculated by three correction algorithms of ISO 7933, ISO 9920, and Lu, with empty spots for *v_a_* = 0.5 m/s, partially filled for the *v_a_* = 1 m/s, and fully filled for the *v_a_* = 3 m/s. * *p* < 0.05.

Figure 1 shows the dynamic clothing insulation values of the three types of clothing using ISO 7933, ISO 9920, and Lu’s algorithm. The dynamic clothing insulation values decreased due to the wind and the body movement, which made the clothing microclimate to form forced convection and improved the heat exchange and reduced the ability of clothing resistance. For NC (*I_cl_* = 0.6, 0.8, 1 clo), the dynamic clothing insulation values calculated, consistent with ISO 9920, appeared to be higher than those obtained by applying ISO 7933 correction formulas, and the maximum difference percentage was 15%, Figure 1a. This discrepancy became more significant as the walking speed or the air velocity increased. For example, the deviation of the two *I_cl,dyn_* values had an increase from 2% to 13% when the walking speed values increased from 0.4 to 1.2 at *v_a_* = 0.5 m/s. However, the effect of air velocity decreased if the walking speed value was high at 1.2 m/s.

For SC (*I_cl_* = 1.5, 2 clo), the dynamic clothing insulation values calculated by ISO 9920 proved still higher than those for ISO 7933, and the maximum deviation between the two *I_cl,dyn_* values was much higher than that in NC, 82%. As the air velocity or the walking speed increased, the discrepancy grew larger from 30% to 82%.

As for LC (*I_cl_* = 0.2, 0.4 clo), most of the dynamic clothing insulation values calculated by ISO 7933 were the highest, the next were the values obtained through the algorithm of Lu, and the lowest were those calculated based on ISO 9920. Nevertheless, there was no significant difference between ISO 7933 and Lu when the basic clothing insulation was 0.2 clo (*p* = 0.146), and the same situation was found between ISO 9920 and Lu when the basic value was 0.4 clo (*p* = 0.157). The difference of the dynamic values using ISO 7933 and ISO 9920 decreased from 47% to 20% with increasing air velocity and walking speed, which was the opposite of the case for NC or SC.

#### 3.1.2. Clothing Dynamic Evaporative Resistance

**Figure 2:**
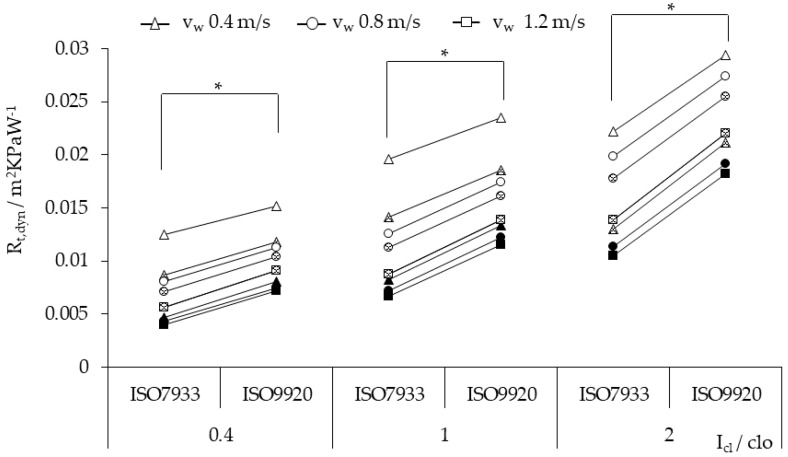
The dynamic evaporative resistance of three types of clothing calculated by two correction algorithms, ISO 7933 and ISO 9920, with empty spots for *v_a_* = 0.5 m/s, partially filled for *v_a_* = 1 m/s, and fully filled for *v_a_* = 3 m/s. * *p* < 0.05.

Three types of clothing each chose a static clothing insulation value for the dynamic evaporative resistance calculation, 0.4 clo for LC, 1 clo for NC, 2 clo for SC. Figure 2 shows that the dynamic evaporative resistance with ISO 7933 equations was lower than that with ISO 9920, and the difference between the two algorithms became more apparent with the increase of static thermal resistance values. The dynamic evaporative resistance increased from 20% to 73% when the walking speed increased.

### 3.2. Predicted Heat Strain

#### 3.2.1. Rectal Temperature

**Figure 3:**
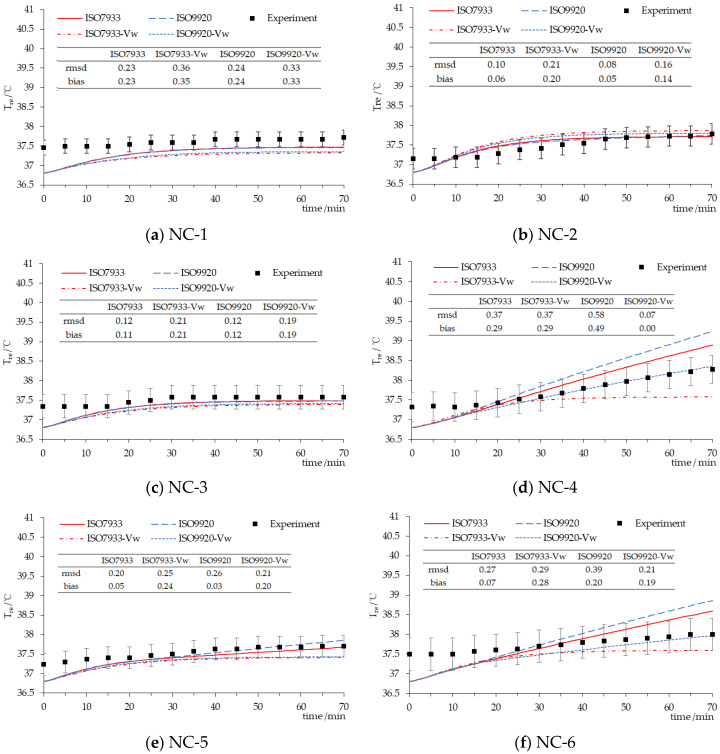
Time-course changes in the rectal temperature for NC. Experiment—the mean rectal temperature observed in participants, ISO7933—the rectal temperature predicted by the PHS model with the clothing correction algorithm of ISO 7933, ISO7933-*v_w_*—the rectal temperature predicted by the PHS model with the clothing correction algorithm of ISO 7933 on adding the walking speed input parameter, ISO9920—the rectal temperature predicted by the PHS model with the clothing correction algorithm of ISO 9920, ISO9920-*v_w_*—the rectal temperature predicted by the PHS model with the clothing correction algorithm of ISO 9920 on adding the walking speed input parameter.(**a**) clothing insulation 0.63 clo in 20 °C; (**b**) clothing insulation 0.63 clo in 40 °C; (**c**) clothing insulation 1.08 clo in 20 °C; (**d**) clothing insulation 1.08 clo in 40 °C; (**e**) clothing insulation 1.11 clo in 20 °C; (**f**) clothing insulation 1.11 clo in 30 °C.

The comparison of experimental data and the predicted values by the PHS model with two different algorithms and two input patterns for rectal temperature while wearing NC ensembles is presented in Figure 3. The rectal temperature predicted by the PHS model with ISO 7933 or ISO 9920 correction formulas did not have a good prediction with experimental data for NC-1 since the rmsd was out of SD (0.19) of the measurement, while the rest of the NC were in the range of SD (0.26, 0.3, 0.36, 0.28, 0.41). Under a 20 °C condition, the predicted rectal temperature was slightly lower than the measured values, according to Figure 3a,c,e. Under the 30 or 40 °C hot conditions, the predicted values by the PHS model with ISO 9920 corrections and adding *v_w_* input parameter were closer to the experimental data according to Figure 3d,f. Moreover, the PHS model only with ISO 7933 and ISO 9920 corrections had a good prediction accuracy for low clothing insulation or a cool environment, according to Figure 3a–c,e.

**Figure 4:**
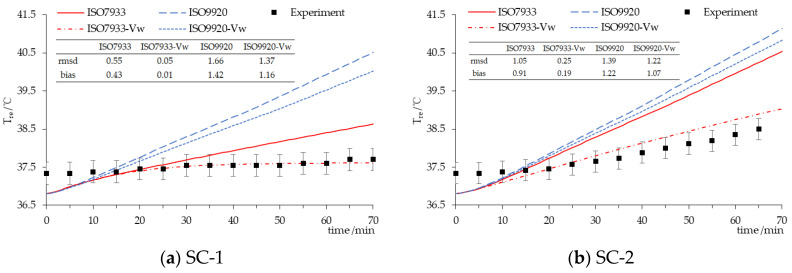
Time-course changes in the rectal temperature for SC. (**a**) clothing insulation 2.01 clo in 30 °C; (**b**) clothing insulation 2.01 clo in 40 °C.

For SC, Figure 4 shows that the prediction accuracies of the rectal temperature by PHS model with ISO 9920 and ISO 7933 corrections were both poor because the rmsd was larger than the SD in the experimental data (0.29, 0.28). In particular, the predicted ending point of the rectal temperature with the ISO 9920 correction formula was 2 °C higher than the observed value. However, if the input parameters of the walking speed were added, the prediction accuracy of the PHS model would be significantly improved. For example, the predicted ending point of the rectal temperature with ISO7993-*v_w_* was only 0.09 °C lower than the experimental data for SC-1.

**Figure 5:**
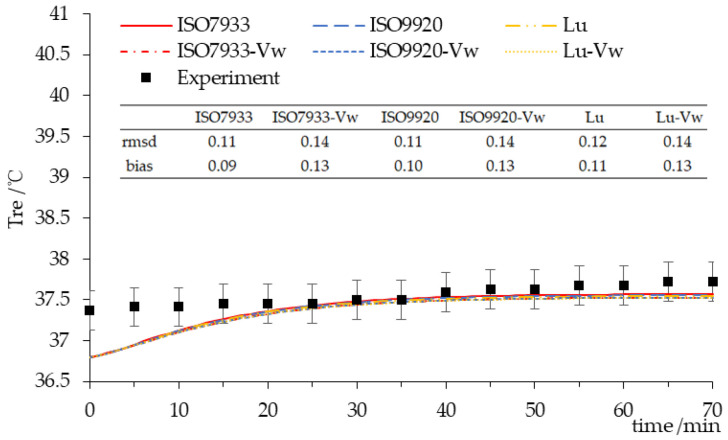
Time-course changes in the rectal temperature for the LC. Lu—the rectal temperature predicted by the PHS model with the clothing correction algorithm of Lu. Lu-*v_w_*—the rectal temperature predicted by the PHS model with the clothing correction algorithm of Lu on adding the walking speed input parameter.

For LC, the results of the rectal temperature prediction with different algorithms are presented in Figure 5. Although these results indicated that the predicted rectal temperature was slightly lower than the observed value, the rmsd of the rectal temperatures predicted by PHS model with ISO 7933, ISO 9920, or the Lu correction formula were far lower than the SD of experimental data (0.24), which meant those three algorithms all had a good prediction accuracy. Among them, the PHS model with ISO 7933 and ISO 9920 was the best. However, the prediction accuracy had not improved yet when adding the walking speed input parameter. For the final rectal temperature, the largest was the value with ISO 7933, and then ISO 9920, followed by Lu with *v_w_*, Lu, ISO 7933 with *v_w_*, and ISO 9920 with *v_w_*.

#### 3.2.2. Water Loss

**Figure 6:**
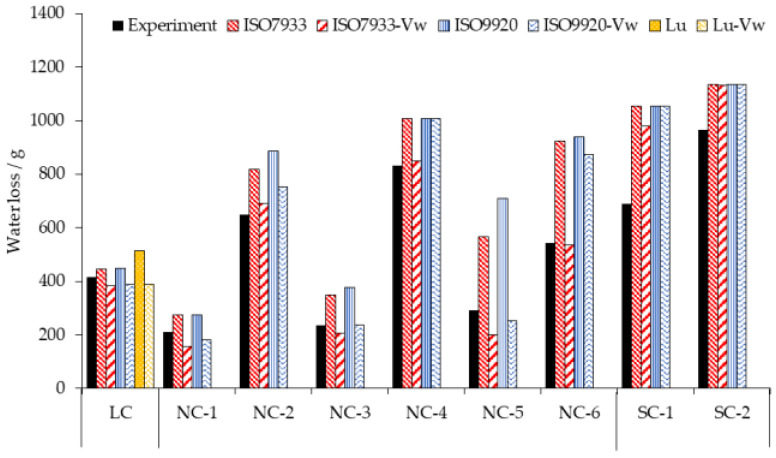
The water loss for three types of clothing observed on participants (Experiment) and calculated by the PHS model with clothing correction algorithm of ISO 7933, ISO 9920, and Lu on adding the walking speed input parameter (ISO7933-*v_w_*, ISO9920-*v_w_*, Lu-*v_w_*).

Figure 6 shows the results of the water loss prediction for three types of clothing in a 70 min time period. The three algorithms all overestimated the water loss of the human body. By comparison, the predicted value of the water loss with ISO 7933 correction formulas was closer to the observed data. While adding the walking speed as an additional parameter in our calculations, the prediction accuracy was significantly enhanced. For example, the predicted values with ISO7933-*v_w_* or ISO9920-*v_w_* dropped and the difference percentage reduced to within 20% except for SC-1. For NC, the prediction accuracy with ISO9920-*v_w_* was better when the air temperature was as low as 20 °C, and ISO7933-*v_w_* proved more accurately when the air temperature was as high as 30 or 40 °C. For SC, the PHS model with ISO 7933 and the addition of the *v_w_* input parameter could improve the prediction accuracy. For LC, the difference in percentage between the predicted values and the observed data was within 10% except for the algorithm of Lu.

#### 3.2.3. Maximum Allowable Exposure Time

According to ISO 7933, the maximum water loss is 5% of the body mass and the rectal temperature is 38 °C for an average subject [2]. Table 6 showed the predicted and observed values of the maximum exposure time of the three types of clothing. For NC-4, PHS with ISO 7933 or ISO 9920 corrections underestimated the maximum exposure time, which was similar to the previous studies [15]. However, when adding the *v_w_* input parameter and using the ISO 9920 corrections, the predicted time was closer to the experimental data. For the rest of the NC, the maximum allowable exposure time was 70 min. For SC, the three algorithms underestimated the maximum exposure time, and the closest to the experimental data was the value predicted with ISO7933-*v_w_*. For LC, the upper limit of human tolerance was not reached within 70 min, and all predictions by three algorithms and the observation were 70 min.

## 4. Discussion

### 4.1. Dynamic Thermophysical Properties of Clothing

By analyzing data summarized in Table 1 and Figure 1a, it can be discovered that 0.4 clo < *I_cl_* ≤ 1 clo. As such, the dynamic clothing insulation values calculated by ISO 9920 corrections were a little higher than those with ISO 7933 corrections, with the discrepancy ranging from 2% to 15%. However, when *I_cl_* ≤ 0.4 clo, the discrepancy between two correction approaches became larger, according to Table 3 and Figure 1c, and this finding was the same as that in the study of Alfano [9]. The reason is not the effect of the values of the dynamic air boundary layer calculated by ISO 7933 or ISO 9920, because *I_a,dyn_* values are in close agreement [9]. In search of the underlying causes behind this, Alfano [9] found that both standards returned very close values if the same interpolation procedure was used. Therefore, the possible reason is the different correction approach of the total insulation, which means that *C_orr__,tot_* (Table 1) used in ISO 9920 is more significant than that in ISO 7933. Furthermore, when *I_cl_* > 1 clo, the discrepancy of the dynamic clothing insulation between ISO 7933 and ISO 9920 also varied largely, according to Table 2 and Figure 1b. This is caused because the correction coefficient values used in Table 2 are larger than that in Table 1.

The discrepancy between the dynamic clothing insulation values calculated by ISO 7933 and ISO 9920 corrections became more substantial with the increase of air velocity and walking speed when *I_cl_* > 0.4 clo, but it was the opposite when *I_cl_* ≤ 0.4 clo. The possible reason may be the effect of the correction approaches of the air boundary layer in the two approaches. The discrepancy of the effective insulation of the air boundary layer between the two standards appears smaller as the air velocity increases [9]. As for light clothing, the total dynamic clothing insulation is easily influenced by the air boundary layer.

For dynamic evaporative resistance, the calculated values with ISO 9920 were larger than those with ISO 7933. Our findings agree with those reported by Ueno [16] for light clothing (*I_cl_* = 0.3 clo) and Alfano [9] for normal and light clothing (*I_cl_* ≤ 1 clo). Ueno [16] confirmed that the difference of approaches was weak, at 5% [16]. Alfano [9] reported that the reason is that ISO 7933 is a double correction, including the correction of basic clothing insulation and permeability index, while ISO 9920 only corrects the static evaporative resistance value.

Due to the propagation of error, the prediction accuracy of heat strain will decrease. Therefore, it is necessary to clarify the scope of application of each correction algorithm, as well as to select the appropriate evaluation approach of dynamic thermophysical properties of clothing to apply in the PHS model.

### 4.2. Heat Strain Predicted by the PHS Model

Three types of clothing were classified and the main physiological variables for each category were predicted and compared with measurement data. For NC, the predicted rectal temperature and the water loss by the PHS model with ISO 9920 correction formulas were larger than those obtained with ISO 7933 corrections, which were the same as in the studies of Alfano [9] and Ueno [16]. It is because the values of thermophysical properties of clothing obtained by ISO 9920 correction formulas are higher. This leads to a decrease of the maximum evaporation rate and a decrease of the predicted evaporation rate, as soon as the maximum wetness is reached, thus bringing the rectal temperature up to the limit value of 38 °C [9]. Consequently, the maximum exposure time predicted by PHS with ISO 9920 corrections greatly reduces. However, using ISO 9920 corrections and adding the walking speed input parameter into the PHS model has an optimal prediction for the rectal temperature if the air temperature is high and the basic insulation is relatively large (Figure 3). Although the prediction accuracy of the water loss declined under the same condition (Figure 6). As the thirst sensation is not as strong a factor as core temperature, dehydration is harder to notice [17]. That is, compared with Dlimloss, the maximum exposure time is mainly determined by Dlimtre. Therefore, we suggest that the PHS model with ISO 9920 corrections and the addition of the *v_w_* input parameter could be used for a hot environment and high clothing insulation. ISO 7933 corrections should be reserved for other conditions.

For SC, this is the first time to discuss the most effective correction algorithm of the thermophysical properties of clothing while predicted physiological variables by the PHS model. According to Figure 4 and Figure 6, and Table 6, the PHS model with ISO 7933 corrections and the addition of the walking speed input parameter can significantly improve the prediction accuracy of both Tre and water loss. Generally, walking speed does not need to be provided as it can be calculated by the metabolic rate. Adding the walking speed or not will have an impact on the dynamic clothing insulation of 0.58 clo and the evaporative resistance of 0.0309 m^2^·kPa/W^2^ for SC, and thus affect the rectal temperature and water loss prediction by the PHS model. Previous studies did not consider the effect of the walking speed input, so they concluded that the PHS model generated the body core temperature predictions that lay outside reasonable limits when the subjects wore bulky protective clothing [5]. Therefore, it is necessary to increase the input parameters of walking speed to improve the prediction accuracy of the model.

For LC, our data are consistent with the research that the rectal temperature predicted by the PHS model with ISO 7933 corrections slightly underestimated the measurement values and overestimated the maximum exposure time where its basic clothing insulation was 0.32 clo [18]. The final rectal temperature predicted by PHS with ISO 7933 corrections was a little higher than that predicted with ISO 9920. When the basic insulation was small, the dynamic clothing insulation calculated by ISO 7933 corrections was larger than that obtained with ISO 9920, and the dynamic evaporative resistance calculated by ISO 9920 corrections was larger than that by ISO 7933. At this time, the effect of clothing insulation on the PHS prediction procedure exceeds the effect of evaporative resistance, ultimately the rectal temperature predicted by PHS with ISO 7933 corrections was higher. In addition, using ISO 7933 corrections is still more significant than others, because of the lowest rmsd and the lowest prediction error of the water loss, 7%. However, if adding the walking speed input parameter, the accuracy decreases on the contrary. Therefore, we suggest that ISO 7933 corrections should be still employed to light clothing.

The limitation of this study is related to the validation tests, which were restricted from the literature. Further validation is required for both laboratory and field studies covering controlled and uncontrolled environmental conditions and human activities. Another limitation regarding the validation of the maximum allowable time for the light clothing should be considered, and longer exposure duration should be provided for additional developments.

## 5. Conclusions

A comparison of different correction approaches of the thermophysical properties of clothing and the body physiological variables predicted by the PHS model with different corrections was conducted. The following main conclusions were obtained.

(1) The clothing dynamic insulation values calculated by ISO 9920 correction formulas are larger than those obtained by ISO 7933 corrections when *I_cl_* > 0.4 clo. However, when *I_cl_* ≤ 0.4 clo, the calculation results of the dynamic clothing insulation vary contrarily.
(2) The discrepancy between the dynamic clothing insulation values calculated by ISO 7933 and ISO 9920 corrections becomes large with the increase of air velocity and walking speed when *I_cl_* > 0.4 clo, but it is opposite when *I_cl_* ≤ 0.4 clo.
(3) The dynamic evaporative resistance values calculated by ISO 9920 corrections are always larger than those obtained by ISO 7933 corrections.
(4) The clothing dynamic insulation values calculated by the Lu’s corrections are between those obtained by ISO 7933 and ISO 9920. The rectal temperature predicted by the PHS model with Lu’s corrections is good, but the prediction accuracy of the water loss decreases.
(5) According to the comparison between the different prediction values of three clothing corrections with experimental data, ISO 9920 corrections with the addition of the walking speed input parameter can be used for NC for hot environment and high clothing insulation. For the rest of the NC conditions, ISO 7933 correction formulas should still be used. For clothing SC, ISO 7933 corrections with the addition of the walking speed input parameter can improve the prediction accuracy of the PHS model. For LC, it is suggested that ISO 7933 corrections are the best for the PHS model.

## Figures and Tables

**Table 1:** Calculation of *C_orr,tot_, C_orr,a_* coefficient of normal clothing (NC) according to ISO 7933 and ISO 9920. Relative velocity: *v_ar_*, walking speed: *v_w_*_._

	ISO 7933	ISO 9920
*C_orr__,tot_*	$e^{\left(0.043-0.398v_{ar}+0.066v_{ar}{}^{2}-0.378v_{w}+0.094v_{w}{}^{2}\right)}$	$e^{\left[-0.281(v_{ar}-0.15)+0.044{\left(v_{ar}-0.15\right)}^{2}-0.492v_{w}+0.176v_{w}{}^{2}\right]}$
*C_orr,a_*	$e^{\left(-0.472v_{ar}+0.047v_{ar}{}^{2}-0.342v_{w}+0.117v_{w}{}^{2}\right)}$	$e^{\left[-0.533(v_{ar}-0.15)+0.069{\left(v_{ar}-0.15\right)}^{2}-0.462v_{w}+0.201v_{w}{}^{2}\right]}$
Ranges	0 ≤ *v_ar_* ≤ 3 m/s, 0 ≤ *v_w_* ≤ 1.5 m/s	0.15 ≤ *v_ar_* ≤ 3.5 m/s, 0 ≤ *v_w_* ≤ 1.2 m/s

**Table 2:** Calculation of *C_orr__,tot_* coefficient of specialized, insulating, cold weather clothing (SC) according to ISO 9920. Air permeability of clothing: *pr.*

	ISO 9920
*C_orr__,tot_*	$e^{[-0.0512(v_{ar}-0.4)+0.000794{\left(v_{ar}-0.4\right)}^{2}-0.0639v_{w}]pr^{0.144}}$
Ranges	0.4 ≤ *v_ar_* ≤ 18 m/s, 0 ≤ *v_w_* ≤ 1.2 m/s, 1 ≤ *pr* ≤ 1000 L/m^2^s

**Table 3:** Comparison of different approaches and different equations of light clothing (LC) according to ISO 7933, ISO 9920, and the study of Lu.

Approach	Algorithm	Equation	Range
Coefficient	ISO 7933	$I_{tot,dyn}=\frac{(0.6-I_{cl})C_{orr,a}+I_{cl}C_{orr,tot}}{0.6}I_{tot}$	0 ≤ *v_ar_* ≤ 3 m/s
			0 ≤ *v_w_* ≤ 1.5 m/s
Interpolation	ISO 9920	$I_{tot,dyn}=\frac{(0.6-I_{cl})I_{a,dyn}+I_{cl}I_{tot,dres}{}_{s}}{0.6}$	0 ≤ *v_ar_* ≤ 3 m/s
			0 ≤ *v_w_* ≤ 1.5 m/s
Coefficient	Lu	$I_{tot,dyn}=e^{\left[-0.393(v_{ar}-0.15)+0.0393{\left(v_{ar}-0.15\right)}^{2}-0.0728v_{w}+0.053v_{w}{}^{2}\right]}I_{tot}$	0.15 ≤ *v_ar_* ≤ 5.2 m/s
			0 ≤ *v_w_* ≤ 1.2 m/s

**Table 4:** Calculation of the dynamic evaporative resistance according to ISO 7933 and ISO 9920. Permeability index: *i_mt_*, static evaporative resistance: *R_t_.*

Algorithm	Equation
ISO 7933	$i_{mt,dyn}=i_{mt}*C_{orr,E}$ $C_{orr,E}=2.6C_{orr,tot}{}^{2}-6.5C_{orr,tot}+4.9$ $R_{t,dyn}=I_{tot,dyn}/(i_{mt,dyn}/16.7)$
ISO 9920	$R_{t,dyn}=(1.2C_{orr,tot}{}^{2}-0.5C_{orr,tot}+0.3)R_{t}$

**Table 5:** The input parameters of the PHS model for predicted rectal temperature, water loss, and maximum exposure time.

Clothing Type	*I_cl_*	*R_cl_*	*i_mt_*	*Ta*	*Pw*	*v_a_*	*v_w_*	*Met*	*Time*
	(clo)	(m^2^·kPa/W^2^)	(nd)	(°C)	(kPa)	(m/s)	(m/s)	(W/m^2^)	(min)
LC	0.48	0.0198	0.49	30	2.0	0.33	1.25	163	70
NC-1	0.63	0.0257	0.43	20	2.0	0.33	1.25	169	70
NC-2	0.63	0.0257	0.43	40	2.2	0.33	1.25	171	70
NC-3	1.08	0.0421	0.36	20	2.0	0.33	1.25	163	70
NC-4	1.08	0.0421	0.37	40	3.3	0.33	1.25	155	70
NC-5	1.11	0.0745	0.21	20	2.0	0.33	1.25	167	70
NC-6	1.11	0.0745	0.21	30	2.0	0.33	1.25	175	70
SC-1	2.01	0.1224	0.2	30	2.0	0.33	1.25	190	70
SC-2	2.01	0.1224	0.2	40	2.2	0.33	1.25	190	70

**Table 6:** The maximum exposure time for three types of clothing observed on participants (Experiment) and calculated by the PHS model with clothing correction algorithm of ISO 7933, ISO 9920, and Lu on adding the walking speed input parameter (ISO7933-*v_w_*).

Time	NC-4					SC-2					LC						
	Experiment	ISO 7933	ISO7933-*v_w_*	ISO 9920	ISO9920-*v_w_*	Experiment	ISO 7933	ISO7933-*v_w_*	ISO 9920	ISO9920-*v_w_*	Experiment	ISO 7933	ISO7933-*v_w_*	ISO 9920	ISO9920-*v_w_*	Lu	Lu-*v_w_*
Dlimtre ^1^	50	40	70	35	52	45	25	37	23	24	70	70	70	70	70	70	70
Dlimloss95 ^2^	70	70	70	70	70	70	70	70	70	70	70	70	70	70	70	70	70
Maximum time	50	40	70	35	52	45	25	37	23	24	70	70	70	70	70	70	70

Note: ^1^ Dlimtre is the maximum time determined by rectal temperature, ^2^ Dlimloss95 is the maximum time determined by water loss.
